# Current status of boron neutron capture therapy of high grade gliomas and recurrent head and neck cancer

**DOI:** 10.1186/1748-717X-7-146

**Published:** 2012-08-29

**Authors:** Rolf F Barth, M Graca H Vicente, Otto K Harling, WS Kiger, Kent J Riley, Peter J Binns, Franz M Wagner, Minoru Suzuki, Teruhito Aihara, Itsuro Kato, Shinji Kawabata

**Affiliations:** 1 Department of Pathology, The Ohio State University, 165 Hamilton Hall, 1645 Neil Avenue, Columbus, OH, 43210, USA; 2 Department of Chemistry, Louisiana State University, Baton Rouge, LA, 70803, USA; 3 Department of Nuclear Science & Engineering, Massachusetts Institute of Technology, Cambridge, MA, 02139, USA; 4 Department of Radiation Oncology, Beth Israel Deaconess Medical Center, Harvard Medical School, Boston, MA, 02215, USA; 5 Department of Radiation Oncology, Massachusetts General Hospital, Boston, MA, 02114, USA; 6 Department of Radiation Oncology, Mt. Auburn Hospital, Cambridge, MA, 02138, USA; 7 Forschungs-Neutronenquelle Heinz Maier-Leibnitz (FRM II), Technische Universität München, Garching, Germany; 8 Particle Radiation Oncology Research Center, Kyoto University, Osaka, Japan; 9 Department of Otolaryngology and Head and Neck Surgery, Kawasaki Medical School, Okayama, Japan; 10 Department of Oral and Maxillofacial Surgery II, Graduate School of Dentistry, Osaka University, Osaka, Japan; 11 Department of Neurosurgery, Osaka Medical College, Takatsuki City, Osaka, Japan

**Keywords:** Boron neutron capture therapy, Gliomas, Head and neck cancer, Radiation therapy

## Abstract

Boron neutron capture therapy (BNCT) is a biochemically targeted radiotherapy based on the nuclear capture and fission reactions that occur when non-radioactive boron-10, which is a constituent of natural elemental boron, is irradiated with low energy thermal neutrons to yield high linear energy transfer alpha particles and recoiling lithium-7 nuclei. Clinical interest in BNCT has focused primarily on the treatment of high grade gliomas, recurrent cancers of the head and neck region and either primary or metastatic melanoma. Neutron sources for BNCT currently have been limited to specially modified nuclear reactors, which are or until the recent Japanese natural disaster, were available in Japan, the United States, Finland and several other European countries, Argentina and Taiwan. Accelerators producing epithermal neutron beams also could be used for BNCT and these are being developed in several countries. It is anticipated that the first Japanese accelerator will be available for therapeutic use in 2013. The major hurdle for the design and synthesis of boron delivery agents has been the requirement for selective tumor targeting to achieve boron concentrations in the range of 20 μg/g. This would be sufficient to deliver therapeutic doses of radiation with minimal normal tissue toxicity. Two boron drugs have been used clinically, a dihydroxyboryl derivative of phenylalanine, referred to as boronophenylalanine or “BPA”, and sodium borocaptate or “BSH” (Na_2_B_12_H_11_SH). In this report we will provide an overview of other boron delivery agents that currently are under evaluation, neutron sources in use or under development for BNCT, clinical dosimetry, treatment planning, and finally a summary of previous and on-going clinical studies for high grade gliomas and recurrent tumors of the head and neck region. Promising results have been obtained with both groups of patients but these outcomes must be more rigorously evaluated in larger, possibly randomized clinical trials. Finally, we will summarize the critical issues that must be addressed if BNCT is to become a more widely established clinical modality for the treatment of those malignancies for which there currently are no good treatment options.

## Introduction

Boron neutron capture therapy (BNCT) is based on the nuclear capture and fission reactions that occur when boron-10, which is a non-radioactive constituent of natural elemental boron, is irradiated with low energy (0.025 eV) thermal neutrons. This results in the production of high linear energy transfer (LET) alpha particles (^4^He) and recoiling lithium-7 (^7^Li) nuclei, as shown below.

In order to be successful, a sufficient amount of ^10^B must be selectively delivered to all tumor cells (~ 20 μg/g weight or ~10^9^ atoms/cell), and enough thermal neutrons must be absorbed to cause lethal damage from the ^10^B(n,α)^7^Li capture reaction [1]. The destructive effects of these high energy particles are limited to boron containing cells and since they have very short pathlengths in tissues (5–9 μm), in theory BNCT provides a way to selectively destroy malignant cells and spare adjacent normal cells. Clinical interest in BNCT has focused primarily on high grade gliomas [1-3], and more recently on patients with recurrent tumors of the head and neck region [4-7] who have failed conventional therapy. BNCT primarily is a biochemically rather than a physically targeted type of radiation therapy, and, therefore, it should be possible to selectively destroy tumor cells dispersed in normal tissue, providing that sufficient amounts of ^10^B and thermal neutrons are delivered to the site of the tumor. In this report, we will provide an update on BNCT, as it relates to the treatment of high grade gliomas and cancers of the head and neck region. We will briefly summarize current developments in the design and synthesis boron delivery agents, neutron sources, clinical dosimetry, treatment planning techniques, and past and ongoing clinical trials in the United States, Japan and Europe and finally, critical issues that must be addressed if BNCT is to be successful. Readers interested in more in-depth coverage of these and other topics related to BNCT are referred to the Proceedings of 13^th^ and 14^th^ International Congresses on Neutron Capture Therapy [8,9].

## Boron delivery agents

### General requirements

Research in the area of development of boron-containing delivery agents for BNCT started ~ 50 years ago with the investigation of a large number of low molecular weight boron compounds, from which the first generation agents emerged. The most important requirements for a BNCT delivery agent are: 1.) low toxicity and normal tissue uptake, with a tumor:normal tissue and tumor:blood (T:Bl) boron concentration ratios of ~3; 2.) tumor boron concentration of ~20 μg ^10^B/g tumor; 3.) relatively rapid clearance from blood and normal tissues, and persistence in tumor during neutron irradiations. The only two BNCT delivery agents currently used in clinical trials are sodium mercaptoundecahydro-*closo*-dodecaborate (Na_2_B_12_H_11_SH), commonly known as sodium borocaptate (BSH), and the boron-containing amino acid (L)-4-dihydroxy-borylphenylalanine, known as boronophenylalanine or BPA [10]. Neither of these agents adequately fulfills the criteria indicated above, and for this reason third generation agents incorporating one or more polyhedral borane anions or carboranes have been investigated. With the development of new synthetic techniques and increased awareness of the biochemical requirements needed for effective boron containing agents and their modes of delivery, a number of new boron agents have emerged. The major challenge in the development of such agents has been the requirement for selective tumor cell targeting and the delivery of therapeutic boron levels with minimal normal tissue toxicity. The localized and effective destruction of glioblastoma (GBM) cells in the presence of normal brain represents a greater challenge than for malignancies at other anatomic sites. This is due to an additional biological barrier, the blood–brain barrier (BBB), and the highly infiltrative nature of glioma cells and their molecular heterogeneity.

### Third generation boron delivery agents

Recent efforts to improve the selectivity of boron delivery agents has involved incorporating them into tumor-targeting molecules, such as peptides, proteins, antibodies, nucleosides, sugars, porphyrins, liposomes and nanoparticles. A compilation of third generation delivery agents of low and high molecular weight is summarized in Table 1 and a recent comprehensive review on this topic [11]. The low molecular weight boron delivery agents include boronated natural amino acids (i.e., BPA derivatives with higher percentage of boron by weight) as well as boronated derivatives of other amino acids, such as aspartic acid, tyrosine, cysteine, methionine and serine [12-14]. Additionally, boron-containing unnatural amino acids also have been investigated because of their higher metabolic stability compared with the natural ones. The boronated derivatives of 1-aminocyclobutane-1-carboxylic acid and 1-amino-3-boronocyclopentanecarboxylic acid (designated ABCPC) are examples of such compounds [13,14]. Boron-containing linear and cyclic peptides are being investigated because they are usually non-immunogenic, easy to synthesize, and often show low toxicity and high tissue penetrating properties [15]. Of particular interest are peptide ligands for over-expressed receptors on tumor cells, such as the vascular endothelial growth factor receptor (VEGFR), somatostatin receptors and the epidermal growth factor receptor (EGFR), enzyme substrates and intracellular delivery sequences.

**Table 1:** Examples of new low- and high-molecular-weight boron delivery agents currently under evaluation*

**Boronated unnatural amino acids **[12-14]	**Carboranyl nucleosides **[16,17]
Dodecaborate cluster lipids and cholesterol derivatives [18]	Boronated porphyrins [19-21]
Boron containing immunoliposomes [22] and liposomes [23-26]	Boronated EGF and anti-EGFR and VEGFR MoAbs [27-33]
Boronated DNA intercalators [34]	Boron-containing nanoparticles [35,36]
Transferrin–polyethylene glycol (TF–PEG) liposomes [37,38]	Carboranyl porphyrazines [39]
Dodecahyrdo-closo-dodecaborate clusters [18]	Boronated cyclic peptides [15]
	Boron nitride nanotubes [40]

* The numbers indicated in brackets are cited in the reference section.

Boron-containing purines, pyrimidines, thymidines, nucleosides and nucleotides have also been investigated as BNCT delivery agents [16,17,41], in particularly 3-carboranyl thymidines (3CTAs). For example, the thymidine derivative designated N5-2OH displays selective tumor uptake, a high rate of phosphorylation and low toxicity [16]. Convection enhanced delivery (CED) of N5-2OH to rats bearing intracerebral RG2 gliomas has been effective for the selective delivery of therapeutic concentrations of boron to tumors with very high tumor:brain and tumor:blood ratios without any concomitant toxicity [17].

Boron-containing porphyrin derivatives (porphyrins, chlorins, bacteriochlorins, tetrabenzoporphyrins, and phthalocyanines) have been extensively investigated due to their usually low toxicity and natural affinity for tumors [19,20]. Examples of such compounds are BOPP [21], CuTCPH, and H_2_OCP. Porphyrin derivatives have been shown to deliver therapeutic amounts of boron to tumor bearing mice and rats. They attained high tumor:brain and tumor:blood boron concentration ratios, and longer retention times in tumors than BSH and BPA. In addition, boron-containing chlorins, bacteriochlorins and phthalocyanines are promising dual agents for both BNCT and photodynamic therapy (PDT) of tumors. Two of these porphyrin derivatives have been FDA-approved as photosensitizers for PDT. This is due to the strong absorptions of these macrocycles in the red and near-infrared regions of the optical spectrum, and their unique photosensitizing properties [19]. Other boron-containing DNA binding molecules, including alkylating agents, DNA intercalators, minor-groove binders and polyamines have been investigated [34]. For example, derivatives of aziridines, acridines, phenanthridines, various Pt(II) complexes and carboranylpolyamines have been described. These compounds sometimes show low tumor selectivity and significant toxicity, in part due to their multiple cationic charges and/or ability for binding to DNA of normal cells. Boron-containing sugars, including derivatives of glucose, mannose, ribose, galactose, maltose and lactose have also been investigated [11,42]. This class of molecules usually displays low toxicity and low tumor uptake, in part due to their hydrophilicity and fast clearance from tissues.

Among the high molecular weight boron delivery agents, monoclonal antibodies (MoAbs), liposomes and nanoparticles are the most common. MoAbs are a very promising class of tumor-targeting agents due to their high specificity for molecular targets such as EGFR and VEGFR [27-30]. Extensive studies have been carried out by Barth, Wu and Yang and their co-workers using a heavily boronated precision macromolecule that has been linked by means of heterobifunctional reagents to either the EGFR targeting MoAb cetuximab (Erbitux^TM^) [28], the EGFRvIII targeting MoAb L8A4 [31] or EGF itself [32]. These nanovehicles (NVs) have been administered intracerebrally (i.c.) by means of convection enhanced delivery (CED) to rats bearing receptor positive F98 gliomas, followed by BNCT [28,29,31-33]. The best survival data were obtained in F98 glioma bearing rats when these NVs were combined with intravenous or intra-arterial administration of BPA and BSH [28,29,31,33] yielding a 2–3 fold increase in mean survival times (MST) compared to irradiated controls.

Immunoliposomes can deliver low molecular weight hydrophobic agents, such as BSH, via incorporation into their lipid bilayers [22,23]. Liposomes can also transport large numbers of boronated molecules intracellularly and normally show high tumor boron uptake [24]. Hawthorne, Feakes and their co-workers have extensively investigated liposomes as BNCT delivery agents of a variety of polyhedral boron anions and these studies were recently reviewed [25]. High tumor boron concentrations were attained *in vitro* when polyhedral boron anions are encapsulated in tumor-selective unilamellar liposomes, but *in vivo* their therapeutic efficacy has yet to be demonstrated. Linkage of boron-containing liposomes to the MoAb cetuximab resulted in specific molecular targeting of the immunoliposomes to EGFR expressing F98 glioma cells [22]. Boron-containing lipids bearing covalently-bound boron clusters also have been reported. These nanoparticles have the advantages of showing no leakage of encapsulated boronated compounds, delivering high concentrations of boron to tumor-bearing mice, and increasing survival times following BNCT [26,37]. Additional classes of boron-containing nanoparticles have been investigated as BNCT delivery agents [35,40] and this work has been recently reviewed [36].

### Neutron sources for BNCT

As described in more detail later in this review, clinical studies on BNCT originated at the Brookhaven National Laboratory (BNL) and the Massachusetts Institute of Technology (MIT) in collaboration with the Massachusetts General Hospital (MGH) approximately 60 years ago [2]. The initial trials for the treatment of high grade gliomas were carried out with fission reactor-produced beams of thermal neutrons. The inadequate penetration of tissue by thermal neutrons, 3-4 cm, led the clinical team in the MIT/Harvard trials to introduce intra-operative, open cranium irradiations with an air-filled balloon inserted into the surgical cavity to increase neutron penetration and avoid excessive dose to the scalp. After a hiatus of almost 25 years, more recent clinical trials were initiated at MIT and BNL, in the early 1990’s, using for the first time higher energy neutrons in the epithermal energy range (~0.4 eV ≤ E ≤ 10 keV). These higher energy neutrons obviated the need for intra-operative BNCT when treating deep seated malignancies. Epithermal neutrons, for example, can reach tumors at the midline of the brain at a depth of ~8 cm with therapeutic ratios >1. Epithermal neutrons are now generally used in BNCT irradiations although some intra-operative irradiations with thermal neutrons are still performed. Reactor-based facilities in Japan are capable of producing various mixtures of thermal and epithermal neutron spectra, which can be advantageous for head and neck cancers where deep beam penetration may not be required.

### Fission reactor sources for NCT

The general characteristics of neutron beams for NCT are described in detail elsewhere [43] and the requirements for a facility capable of high patient through-put have been discussed by Harling [44]. Two approaches have been used for the design of epithermal neutron irradiation facilities at fission reactors. Direct use of the core neutrons as the source has been the predominant approach for modification or conversion of existing reactors for NCT. More than eight such facilities have been constructed for clinical use in the Americas, Europe and Asia and several more are under construction. Performance characteristics for many of these facilities have been summarized in a review by Harling and Riley [45]. An interesting new facility, which makes direct use of core neutrons from a low power reactor, was recently constructed in China and is located in a suburb of Beijing [46,47]. This is the first reactor designed specifically for NCT and is based on an ultra-safe, low cost design specially suited for urban populated areas or within a hospital.

**Figure 1:**
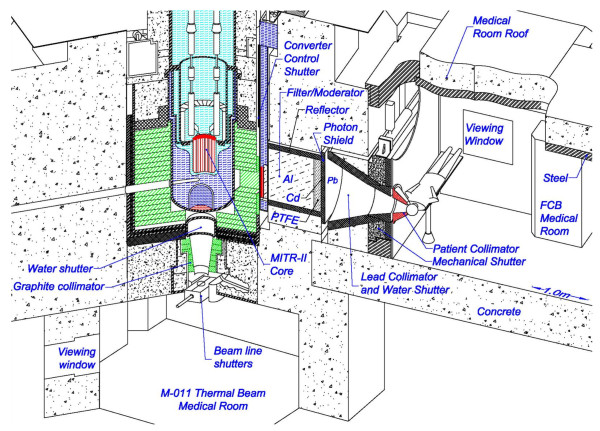
**Schematic diagram of the Massachusetts Institute of Technology Reactor (MITR).** The fission converter based epithermal neutron irradiation (FCB) facility is housed in the experimental hall of the MITR and operates in parallel with other user applications. The FCB contains an array of 11 MITR-II fuel elements cooled by forced convection of heavy water coolant. The converter power is 120 kW at 6 MW reactor power. A shielded horizontal beam line contains an aluminum and Teflon® filter-moderator to tailor the neutron energy spectrum into the desired epithermal energy range. A patient collimator defines the beam aperture and extends into the shielded medical room to provide circular apertures ranging from 16 to 8 cm in diameter. The in-air epithermal flux is 6.2 × 10^9^ n/cm^2^s at the patient position with the 12 cm collimator. The measured specific absorbed doses are constant for all field sizes and are well below the inherent background of 2.8 × 10^-12^ Gy_w_cm^2^/n produced by epithermal neutrons in tissue. The dose distributions achieved with the FCB approach the theoretical optimum for BNCT. This facility is useful for clinical studies of superficial cancers and small animal studies.

Another approach to using fission reactors is based upon the use of a fission converter which converts a reactor’s thermal neutrons to higher energy fission neutrons. The fission neutrons are then moderated and filtered to produce epithermal neutron beams. This approach can be especially useful to modify existing multipurpose reactors. One fission converter based facility, called the FCB (Figure 1) has been constructed at the MIT Research Reactor (MITR) [48,49]. The MIT FCB represents the current state of the art for epithermal neutron irradiation facilities. It meets all requirements for an epithermal neutron irradiation facility needed for routine high throughput clinical NCT. Features and capabilities of the MIT FCB include high intensity, 10–15 minutes to deliver a tolerance dose per irradiation field, accurate delivery of the prescribed neutron fluence, low beam contamination from adventitious radiation components and easy beam positioning on any part of the body. Therapeutic ratios above unity are achieved for depths up to 10 cm using the currently available drug, BPA.

Unless BNCT becomes more broadly accepted as a treatment modality for specific cancers, the need to develop additional nuclear reactors remains limited and further construction seems unlikely. New, special purpose reactors like the one in China, which is still not in clinical use, are a possible source of additional neutron irradiation facilities. Additional reactor conversions and modifications also can be undertaken using proven approaches that require very little research and development effort.

### Accelerator based neutron sources (ABNSs)

To date, all BNCT clinical irradiations have used fission reactor neutron sources, however accelerator based neutron sources are under development and may eventually provide another option to fission reactors. Accelerator based neutron sources are being developed for use in NCT with the anticipation of being easier to site in hospitals and license than a dedicated nuclear reactor. Proponents of accelerators also believe that they could be more compact and less expensive than comparable reactor sources. ABNSs for BNCT have been reviewed in detail elsewhere [50,51]. Generally they produce low intensity neutron fluxes compared to reactor sources and this has been a problem for implementation. An increase in intensity by more than an order of magnitude would be necessary if accelerators are to be competitive with the better reactor sources for clinical BNCT. An ABNS recently has been constructed by Sumitomo Heavy Industries and approval for clinical use has been granted by the Pharmaceutical and Medical Safety Bureau of the Japanese Ministry of Health, Labour, and Welfare [52]. It is anticipated that clinical trials will be initiated in 2013 using this ABNS. Finally, an 8 MeV, 10 mA linac system is under construction and this should be completed in two years (A. Matsumura, Personal Communication).

### Clinical dosimetry and treatment planning

Clinical studies in BNCT have thus far been run usually as single cohorts of patients at individual centers spread across the world. These consisted mainly of Phase I/II trials to establish normal tissue tolerance and secondarily to demonstrate some possible therapeutic efficacy. Each center has, by necessity, developed their own methods to measure, calculate and prescribe absorbed doses for these trials through their own extramural research programs, and this limits their applicability to the local center where they were obtained. After more than 50 years, BNCT still remains, for the most part, an experimental modality with no standardized methods for either calibrating the mixed radiation fields employed or for calculating treatment plans that specify the patient prescription. This lack of uniformity hinders comparison of clinical results from different centers because it precludes combining or sharing clinical data on safety and efficacy in any meaningful way, which should be addressed if BNCT is to have a larger clinical role.

The dosimetry of epithermal neutron beams is complicated by the presence of photon and fast neutron dose components in the radiation field, each possessing a markedly different biological effectiveness that must be quantified separately. In order to account for the varying biological effectiveness of the dose components, multiplicative weighting factors have been applied to the individual dose components to allow expression of their sum as a single, total weighted dose (expressed as Gy_w_), which is approximately equivalent in effect to the same dose of photon radiation. These weighting factors generally have been RBE (relative biological effectiveness) factors measured in animal models [53]. A few common dosimetric methods have been developed to determine the principal absorbed dose components contributed by boron and other low energy neutron capture reactions with normal tissue hydrogen and nitrogen. These dose components generally are determined from absolute measurements of the thermal neutron flux obtained by neutron activation analysis of bare and cadmium-covered gold foils [54,55]. Gamma rays emitted by the activated foils are counted using a gamma spectrometer incorporating either a high-purity germanium or sodium iodide detector with a calibration traceable to a national standards laboratory. The measured thermal neutron flux is multiplied by the appropriate kerma coefficient for a given reaction and the assumed weight fraction of boron (or nitrogen) in the tissue that is being irradiated. Similarly, the twin ionization chamber technique [56], developed for fast neutron therapy [57], is often applied as the method of choice for assessing the adventitious dose arising from both the incident and induced photons as well as fast neutrons contaminating low energy neutron beams. Pairs of detectors are employed with different sensitivities to the two radiation types such as A-150 tissue equivalent plastic- and graphite- (or magnesium-) walled ionization chambers.

Treatment planning for NCT differs markedly from that of conventional radiotherapy and in some ways it is significantly more complex, requiring specialized software [58-61]. NCT treatment planning systems rely exclusively on Monte Carlo simulations for dose calculations because of the complex, scatter-dominated nature of neutron transport. The Monte Carlo method simulates transport of neutrons and photons through the patient’s geometry and uses track-length density estimators of neutron and photon flux integrated against energy-dependent kerma coefficients to compute dose. Treatment planning systems require careful validation by evaluating agreement between dose calculations and in-phantom measurements [62,63]. Treatment planning calculations use individualized computational models of the target, constructed from CT or MR images. While electron density is the principal consideration for transport of megavoltage photon beams in tissue, neutron transport is governed by nuclear interactions. Since neutron cross sections vary significantly between elements, NCT dose calculations must model the elemental compositions of different tissue types in the region of interest. Concentrations of hydrogen, nitrogen, and boron have the most influence on neutron transport and dosimetry.

**Figure 2:**
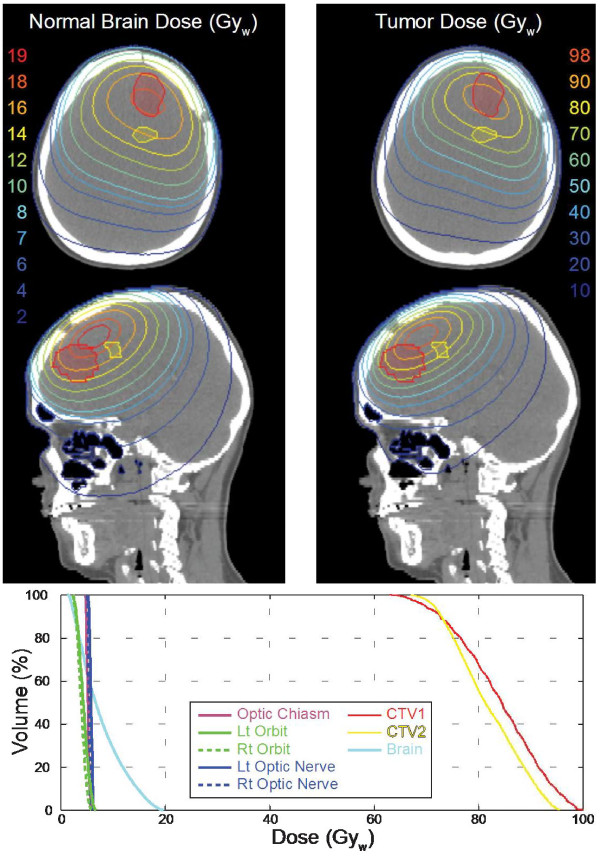
**A three-field treatment plan for a brain tumor (GBM) patient calculated using the MiMMC treatment planning system.** The prescription is a mean brain dose of 7.7 Gy_w_. Isodose contours calculated for tumor and normal brain are shown on axial and sagittal slices through the target volumes. The integral dose volume histograms (DVHs) summarize dosimetry for structures of interest including target volumes and organs at risk.

**Figure 3:**
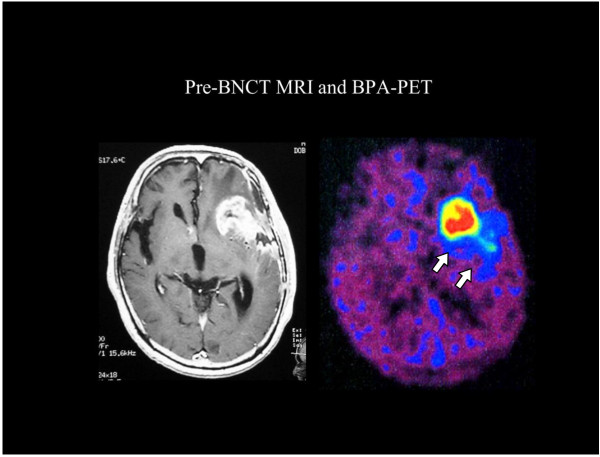
**Contrast-enhanced T1-weighted MRI of representative glioblastoma patient and**^**18**^ **F-labeled BPA-PET image after initial debulking surgery.** The patients received ^18^ F-BPA-PET to assess the distribution of BPA and to estimate the boron concentration in tumors before BNCT without direct determination of boron concentration in the tumor. The lesion to normal brain (L/N) ratio of the enhanced tumor was 7.8 in this case. Note that even the periphery of the main mass, i.e., the infiltrative portion of the tumor, showed BPA uptake. The L/N ratio of BPA uptake can be estimated from this study and dose planning was done according to this L/N ratio, and if the L/N ratio was more than 2.5, then BNCT was initiated. ^18^ F-BPA-PET accurate BPA provided an accurate estimate of the accumulation and distribution of BPA as previously reported [64,65].

**Figure 4:**
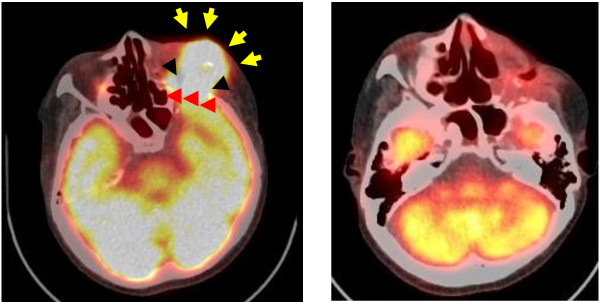
^**18**^**FDG-PET study prior and 6 months after BNCT of a 56 year-old male patient with recurrent squamous cell carcinoma of the maxilla.A**: FDG accumulated in the left orbital region (arrows) and frontal lobe of brain (arrow heads). **B**: No accumulation of FDG-PET was detected 6 months after BNCT and the patient was disease free for 61 months at the time of the original report. Photographs are from *Applied Radiation and Isotopes*, 67:S37-S42, 2009.

BNCT treatment plans are typically presented as total weighted doses, as shown in Figure 2 for tumor and normal tissue(s) and each is calculated separately for the entire dose grid using boron concentrations and weighting factors (RBEs) assumed for each tissue. The rationale for this approach is that the exact boundary of the tumor is unknown and that microscopic deposits of tumor extend well beyond the enhancing volume detectable by imaging. Generally, the tumor and tissue boron concentrations used for dose calculations in treatment planning are assessed as the product of blood boron concentrations measured for the patient at the time of irradiation and static tissue:blood boron concentration ratios determined in previous studies. For example, with BPA-fructose (BPA-F) common assumptions for tissue:blood boron concentration for tumor, normal brain, and skin are 3.5, 1.0, and 1.5. However, as shown in Figures 3 and 4, in cases where ^18^ F]BPA-F PET scans [64] are available, these data often are incorporated into the treatment plan and used to estimate the spatial distribution of boron in dose calculations. Application of ^18^ F]BPA-F PET data to dosimetry must, however, be interpreted with caution because of considerations such as the low spatial resolution and volume averaging effects of the PET scan as well as differences between the bolus injection of radiotracer and the large volume infusion of BPA-F that occurs over an extended time for treatment.

**Figure 5:**
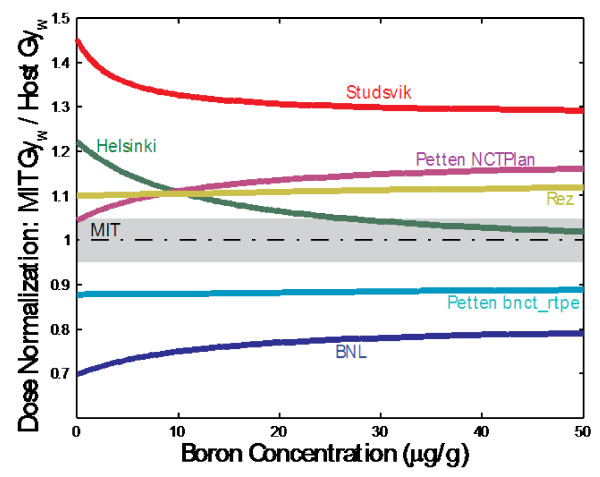
**Graphical representation of a parameterized model converting the maximum weighted dose, calculated Treatment Planning System (TPSs) from each center to an MIT calibrated dose as a function of boron uptake in tissue **[69]. The plotted curves afford an easy and direct comparison of dose specification between participants of the International Dosimetry Exchange.

Although most BNCT centers use some variant of these dosimetry and treatment planning methods, it is important to recognize that while the individual centers achieve satisfactory consistency or precision within particular patient cohorts, dose specifications vary considerably in absolute terms between centers. An extensive series of comparisons was performed between all clinical centers in the Americas and Europe [63,66-69] that found variations ranging between 7.6 and 13.2 Gy_w_ for a common dose specification of 10 Gy_w_ as defined in the Harvard-MIT clinical trials. The magnitude and range of these variations, illustrated in Figure 5 for several facilities, cannot be fully explained by experimental uncertainties or measurement errors. These variations exclude possible systematic differences related to biological weighting factors. Rather, these findings highlight specific implementation problems such as beam source definitions [70] or inexact benchmarking of treatment planning systems [62,71] that only become apparent when comparing between centers. The infrastructure and expense associated with founding and running trials has to date been significant, but even larger and more definitive patient trials are still needed to conclusively demonstrate efficacy, especially in patients with high grade gliomas. This is likely beyond the means of any single center and will only be possible through collaborative clinical trials run by multiple institutions. As a prerequisite, the NCT research community needs to recognize the importance of quantifying and establishing dose uniformity between centers and prioritize these endeavors if larger multi center trials are to succeed.

### Clinical studies of BNCT for brain tumors utilizing thermal neutron beams

The clinical potential of BNCT first was recognized by Locher [72] shortly after the discovery of the neutron. However, it was not until 1951 that the first clinical trials were initiated by Sweet at the Massachusetts General Hospital (MGH) and Brownell at MIT [73] and Farr at the Brookhaven National Laboratory (BNL) in New York [74] using sodium tetraborate (borax), sodium pentaborate, *p-*carboxyphenylboronic acid, or sodium decahydrodecaborate (Na_2_B_10_H_10_) as the sole boron delivery agent. However, since these studies utilized thermal neutron beams they will not be discussed here. Interested readers are referred to a comprehensive review describing these studies in detail [2], as well as reports by Farr [74] and Asbury [75] and their co-workers. Similarly, Hatanaka [76] and later Hatanaka and Nakagawa [77] carried out extensive studies in Japan using BSH as the boron delivery agent [78]. Again these studies also used thermal neutron beams, and therefore they will not be discussed in the present review. However, interested readers are referred to articles of Hatanaka [76] and Nakagawa [77], as well as those of other Japanese neurosurgeons who utilized thermal neutron beams.

### Clinical trials carried out in the United States

Clinical trials were again initiated in the United States utilizing epithermal neutron beams in the 1990s when BNCT was resumed at the BNL Medical Research Reactor (BMRR) [79,80] and at Harvard-MIT using the MITR [81]. In a major step forward, BPA was used as the boron delivery agent and, for the first time, patients were irradiated with a collimated beam of higher energy epithermal neutrons in two fractions on consecutive days. These had greater tissue-penetrating properties than thermal neutrons and were well tolerated. These trials benefited from a number of other technological developments that occurred over the intervening decades: Monte Carlo treatment planning and dosimetry based on tomographic imaging, accurate physical dosimetry techniques, rapid and reliable boron analysis techniques, and an improved understanding of the radiobiology of BNCT. The median survival times of patients that had received a single treatment with BNCT were equivalent to those receiving conventional, fractionated radiation therapy [80,81]. Furthermore, these trials established the safety of the new procedure and this set the stage for the use of BPA in combination with epithermal neutrons as the new standard. This subsequently was adopted by clinicians in Japan and Finland for the treatment of patients with high grade gliomas. Worldwide clinical experience using BNCT with epithermal neutrons to treat high grade gliomas and other brain tumors is summarized in Tables 2 and 3.

**Table 2:** BNCT clinical trials in the United States and Europe using epithermal neutron beams for patients with brain tumors

**Medical institution**	**Neutron source**	**Treatment dates**	**Tumor type & No. of patients**^**a**^	**Boron compound & treatment**^**b**^	**Clinical outcome**^**c**^	**Ref.**
Brookhaven National Laboratory, Upton, NY, USA	Brookhaven Medical Research Reactor, BNL, Upton, NY, USA	1994-1999	GBM 53	BPA 250–330 mg/kg in 2 h	MeST: 12.8 mos.	[53,79,80,82]
					2 y OS: 9.4%	
Beth Israel Deaconess Medical Center, Harvard Medical School, Boston, USA	MIT Research Reactor, Massachusetts Institute of Technology, Cambridge, USA	1996-1999	20 GBM	BPA 250–350 mg/kg in 1.5 h	MeST: 11.1 mos (n = 18)	[53,59,81]
			2 IC MM		2 y OS: 12%	
		2002-2003	6 GBM	BPA 14 g/m^2^ in 1.5 h	NA	[61]
Universitätsklinikum Essen, Essen Germany	High Flux Reactor, JRC Petten, The Netherlands	1997-2002	26 GBM	BSH 100 mg/kg in 1.7 h	MeST: 10.4-13.2 mos.	[83,84]
		2004-2006	4 IC MM (>20 IC mets ea.)	BPA 14 g/m^2^ in 1.5 h	OS: < 3 mos.	[85]
Helsinki University Central Hospital, Helsinki, Finland	FiR-1, VTT Technical Research Centre, Espoo, Finland	1999-2001	30 GBM	BPA 290–500 mg/kg in 2 h	MeST: 11.0-21.9 mos.	[86,87]
		2001-2008	20 rGBM	BPA 290–450 mg/kg in 2 h	MeST: 7 mos. post BNCT	[88]
			2 rAA		1 y OS: 36%	
					2 y OS: 0%	
Faculty Hospital of Charles University, Prague, Czech Republic	LVR-15 Reactor, Nuclear Research Institute Rez, Czech Republic	2000-2002	5 GBM	BSH 100 mg/kg in 1 h	NA	[89]
Nyköping Hospital, Nyköping, Sweden	R2-0 Reactor, Studsvik Medical, Nyköping, Sweden	2001-2003	29 GBM	BPA 900 mg/kg in 6 h	MeST: 17.7 mos.	[90-93]
					2 y OS: 14%	
		2003-2004	1 rMMng	BPA 900 mg/kg in 6 h	OS: 32, 26+ mos. post BNCT	[94]
			1 rMC			
		2001-2005	12 rGBM	BPA 900 mg/kg in 6 h	MeST: 8.7 mos. post BNCT	[95]

^a^Including other disease sites and patients treated off-protocol, the total number of patients treated at some reactors is much greater than reported here, for FiR-1, ~260, for KURR, >107, and for JRR-4, >200 patients. ^b^Treatment is indicated only in cases when it is not solely external beam BNCT. ^c^A range of survival is given in some cases to summarize survival reported for different cohorts.

Abbreviations: *r* recurrent, *GBM* glioblastoma multiforme, *IC MM* intracranial metastatic melanoma, *AA* anaplastic astrocytoma, *MMng* malignant meningioma, *MC* mesenchymal chondrosarcoma, *AOA* anaplastic oligoastrocytoma, *MRM* meningioma related malignancy, *IO-BNCT* intraoperative BNCT, *XRT* external beam radiation therapy (photons), *MeST* median survival time, *2 y OS* 2 year overall survival, *RI* radiographic improvement, *NA* not available.

### Clinical trials in Japan

**Figure 6:**
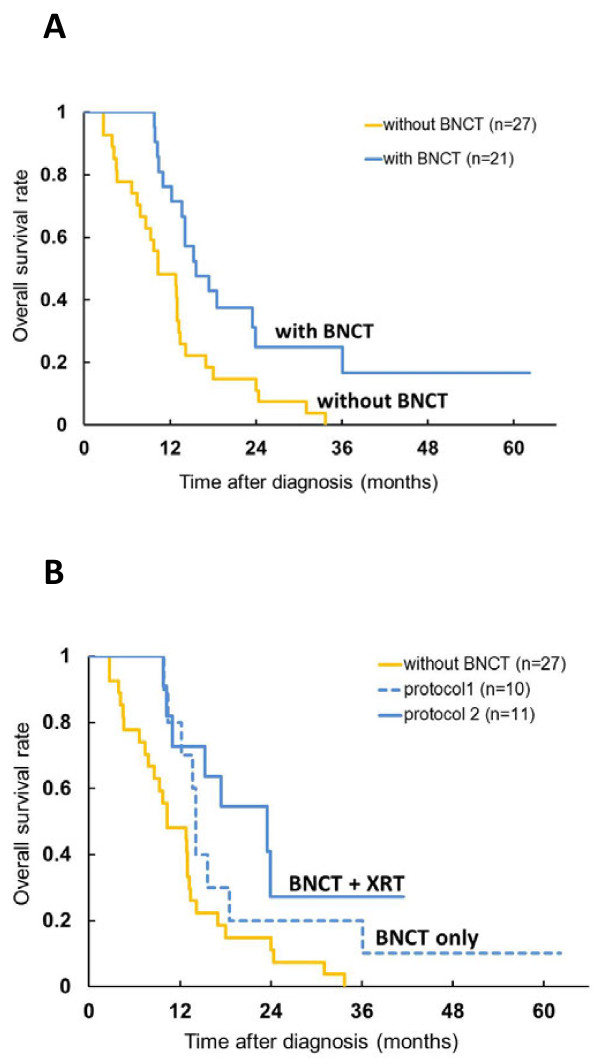
**A. Kaplan-Meier estimates of overall survival for all newly diagnosed glioblastoma (WHO grade 4, n = 21).** The median survival time of boron neutron capture therapy (BNCT) group (blue line) is 15.6 months. There is statistical significance between both group Log-rank test (p = 0.0035). **B.** Kaplan-Meier estimates of overall survival for all newly diagnosed glioblastoma (protocol 1 and 2). External beam X-ray irradiation (XRT) boost after boron neutron capture therapy (BNCT) was carried for the latter 11 cases. This improved the median survival time to 23.5 months (from 14.1 months for BNCT only, protocol 1, dotted line in blue).

**Figure 7:**
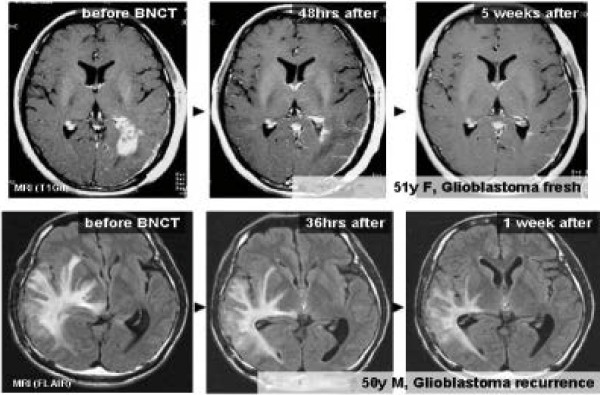
**Radiographic changes following BNCT in two representative patients with GBM.** In both, there was a reduction in both mass and peritumoral edema without the administration of corticosteroids or mannitol within a few days. This is also shown in the FLAIR image of Case #12.

Kawabata and Miyatake and their clinical team have carried out several studies in which either BPA alone or in combination with BSH have been used for BNCT [101-103,106]. The patients had either primary, surgically resected gliomas or recurrent GBMs who had previously received X-irradiation. Favorable responses were seen in patients with newly diagnosed GBMs, especially those in high risk groups [102], using BPA and BSH either with or without an X-ray boost. Patients treated with surgery and BNCT using 100 mg/kg BSH infused over 1 h and 700 mg/kg BPA infused over 6 h followed by 20-30 Gy of fractionated X-rays (Protocol 2) had a median survival time (MeST) of 23.5 months compared to 14.1 mos. for those who had surgery followed by BNCT using 100 mg/kg BSH and a lower dose of BPA, 250 mg/kg infused over 1 h (protocol 1) (Figure 6). The composite MeST of patients who had surgery and BNCT (with or without X-rays) (n = 21), was 15.6 mos. (95% confidence interval (CI): 12.2-23.9 mos. This was significantly longer than 10.3 mos. for historical controls (n = 27) at the Osaka Medical College who had surgery followed by radiation therapy and chemotherapy with ACNU [106]. These clinical studies have validated the animal model data of Barth et al. showing an increase in MSTs of F98 glioma bearing rats by combining BNCT with X-irradiation [107]. Predicting which patients might be more likely to respond to BNCT would be a major step forward. Fluorine-18 labeled BPA has been used to predict the effectiveness of BNCT [64,65], as well as to detect post treatment radiation effects [65]. Representative radiographic changes seen in two patients, treated by BNCT, are shown in Figure 7 and PET imaging studies with ^18^ F-BPA are shown in Figure 3. Although retrospective comparisons must be interpreted with caution, the MeST from the date of diagnosis, calculated using the Kaplan-Meier method (Figure 6), for historical controls was 10.3 months (95% CI: 7.4-13.2, log-rank test p = 0.0035) compared to 23.5 months (95% CI: 10. 2 mos. to undetermined) after diagnosis (n = 11), for patients treated with BNCT plus X-irradiation. However, the MeST of patients in Protocol 1 (BNCT-XRT) was 14.1 months (95% CI: 9.9 mos.-18.5 mos.), which was not significantly different from historical controls, but this may be attributable to low patient numbers since they were subdivided into two protocols involving only 10 and 11 patients each. Kawabata, Miyatake and their co-workers plan to evaluate BNCT in combination with temozolomide in a multicenter Phase II Japanese clinical study (OSAKA-TRIBRAIN0902, NCT00974987). Finally, extensive studies also have been carried out by Matsumura and his clinical team at the University Hospital of Tsukuba [108-110] using either BPA or BSH alone or in combination as the boron delivery agents (see Table 3). In some cases BNCT was combined with a photon boost in combination with temozolomide in patients with either primary or recurrent GBMs. The approach used mosst recently at the University of Tsukuba, using BPA in combination with BSH, followed by a fractionated photon boost, has resulted in the most favorable survival achieved with BNCT to date: 27.1 mos. MeST with a 2 year OS of 63% (n = 8)[109,110]. However, these results must be interpreted, with caution due to the very small number of patients that have been treated. A randomized Phase II clinical trial with a sufficient number of patients will be required to validate these very encouraging but preliminary results.

**Table 3:** BNCT clinical trials in Japan using epithermal or mixed thermal & epithermal neutron beams for patients with brain tumors

**Medical institution**	**Neutron source**	**Treatment dates**	**Tumor type & No. of patients**^**a**^	**Boron compound & treatment**^**b**^	**Clinical outcome**	**Ref.**
University of Tsukuba, Tsukuba City, Ibaraki, Japan	JRR-4, Japan Atomic Energy Agency, Tokai, Ibaraki, Japan	1999-2002	5 GBM	BSH 100 mg/kg in 1–1.5 h	MeST: 23.2 mos. (GBM)	[96]
			4 AA	IO-BNCT	MeST: 25.9 mos. (AA)	
		1998-2007	7 GBM	BSH 5 g in 1 h, IO-BNCT	MeST: 23.3 mos.	[97]
					2 y OS: 43%	
		1998-2007	8 GBM	BSH 5 g in 1 h & BPA 250 mg/kg in 1 h	MeST: 27.1 mos.	[97]
					2 y OS: 63%	
				BNCT + XRT		
University of Tokushima, Tokushima, Japan	JRR-4 or KURR (Kyoto University Research Reactor, Osaka, Japan)	1998-2000	6 GBM	BSH 64.9-178.6 mg/kg	MeST: 15.5 mos.	[98-100]
				IO-BNCT	2 y OS: 0%	
		2001-2004	11 GBM	BSH 64.9-178.6 mg/kg	MeST: 19.5 mos.	[98-100]
				IO-BNCT	2 y OS: 27%	
		2005-2008	6 GBM	BSH 100 mg/kg & BPA 250 mg/kg	MeST: 26.2 mos.	[98-100]
					2 y OS: 50%	
				BNCT + XRT		
Osaka Medical College, Osaka, Japan	KURR	2002-2003	10 GBM	BSH 5 g & BPA 250 mg/kg in 1 h	MeST: 14.5 mos.	[101,102]
					2 y OS: 20%	
		2003-2006	11 GBM	BSH 5 g & BPA 700 mg/kg in 6 h	MeST: 23.5 mos.	[102]
					2 y OS: 27.3%	
				BNCT + XRT		
		2002-2007	19 rGBM	BSH 100 mg/kg & BPA 250 mg/kg in 1 h or	MeST: 10.8 mos. post BNCT	[103]
			2 rAA, 1 rAOA			
				BSH 100 mg/kg & BPA 700 mg/kg in 6 h	2 y OS: 14%	
		2005-2006	7 rMRM	BSH 0–5 g	RI: 100%	[104,105]
				BPA 500–700 mg/kg in 3–4 h		

^a^Including other disease sites and patients treated off-protocol, the total number of patients treated at some reactors is much greater than reported here, for FiR-1, ~260, for KURR, >107, and for JRR-4, >200 patients. ^b^Treatment is indicated only in cases when it is not solely external beam BNCT.

Abbreviations: *r* recurrent, *GBM* glioblastoma multiforme, *IC MM* intracranial metastatic melanoma, *AA* anaplastic astrocytoma, *MMng* malignant meningioma, *MC* mesenchymal chondrosarcoma, *AOA* anaplastic oligoastrocytoma, *MRM* meningioma related malignancy, *IO-BNCT* intraoperative BNCT, *XRT* external beam radiation therapy (photons), *MeST* median survival time, *2 y OS* 2 year overall survival, *RI* radiographic improvement, *NA* not available.

### Clinical studies in Finland

Kankaanranta et al. at the Helsinki University Central Hospital and VTT Technical Research Center of Finland have reported on 22 patients with GBMs who had undergone standard therapy, recurred, and subsequently received BNCT at the time of their recurrence using BPA as the boron delivery agent [88]. The overall MeST was 7 mos. and the median time to progression was 3 mos. However, it is difficult to compare these survival data and those reported for patients with recurrent GBMs receiving conventional treatments. Nevertheless, they are a starting point for future studies using BNCT as salvage therapy in patients with recurrent tumors.

### EORTC clinical study

APhase I clinical trial was carried out by the European Organization for Research and Treatment of Cancer to evaluate BSH as a boron delivery agent (protocol #11961) using the High Flux Reactor at Petten, the Netherlands. The aims of this study were to investigate the systemic toxicity of i.v. administration of BSH, the maximum tolerated radiation dose and the dose limiting toxicity of BNCT. At the time of reporting [83,84] a total of 26 patients had been treated with BNCT with a starting dose of 8.6 Gy. In all but one patient, BNCT was performed in 4 fractions on 4 consecutive days. On the day prior to the first irradiation BSH (100 mg/kg b.w.) was administered i.v. Both the amount administered and the time from BSH infusion to irradiation (range 8–14 h) were adjusted to achieve a blood boron concentration of ~ 30 μg/g during irradiation. The MeST of the first patient group was 10.4 months after the first surgery, 11.3 months for the second group and 13.2 months for the third patient group [83]. However, dose limiting toxicities were observed in this study [84].These mainly consisted of cerebral atrophy and white matter abnormalities [84].

### Clinical studies in Sweden

We would like to conclude this section on BNCT of high grade gliomas with a summary of a clinical trial that was carried out in Sweden using BPA and an epithermal neutron beam. This study differed significantly from all previous clinical trials in that the total amount of BPA infused was increased to 900 mg/kg b.w., and it was given i.v. over 6 hours [90]. This regimen was based on animal studies in F98 glioma bearing rats that showed that boron concentrations in invading tumor cells increased from 37% to 71% of that in the main tumor if the infusion time was increased from 2 h to 6 h [111]. The higher dose and longer infusion time of the BPA were well tolerated by the 29 patients who were enrolled in this study [90-93,112]. The minimum dose to the tumor ranged from 15.4 to 54.3 Gy_w_ and the mean weighted dose to whole brain was 3.2-6.1 Gy_w_ and all were treated with 2 fields. There has been some disagreement among the Swedish investigators who carried out this study on evaluation of the results. One group reported survival data that was compiled before all of the patients had succumbed to their tumors [91]. Based on all of the survival data, another group [92,93,112] determined that the MeST was 17.7 mos. compared to 15.5 mos. for patients who received standard therapy of surgery, followed by radiotherapy (RT) and temozolomide. Furthermore, the frequency of adverse events were lower after BNCT (14%) than after RT alone (21%) and both of these were lower than those seen following RT in combination with temozolomide. If these improved survival data, using a 6-hour infusion time and a higher dose of BPA, can be confirmed by others, preferably in a randomized clinical trial, it could represent a significant step forward in BNCT of brain tumors, especially if combined with a photon boost.

## Clinical studies of BNCT for head and neck cancer

### Studies carried out in Japan

**Figure 8:**
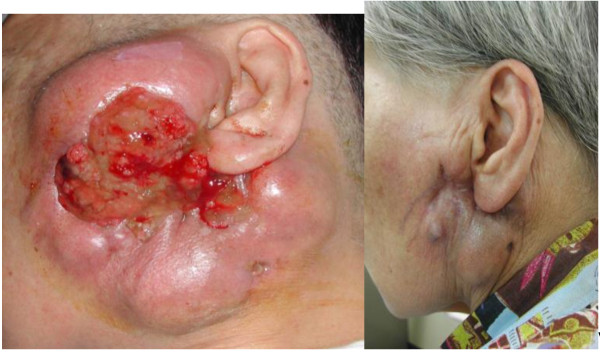
**Before BNCT (left) and 22 months after the first BNCT (right) of a patient with a recurrent mucoepidermoid carcinoma of the parotid gland.** Three treatments with BNCT produced a remarkable reduction in tumor size, but also resolution of a cutaneous ulcer and re-epithelization by normal skin. These results clearly demonstrate that BNCT is a highly tumor-selective treatment modality. She lived for 7 years following treatment (*Applied Radiation and Isotopes,* 61:1069–1073, 2004).

**Figure 9:**
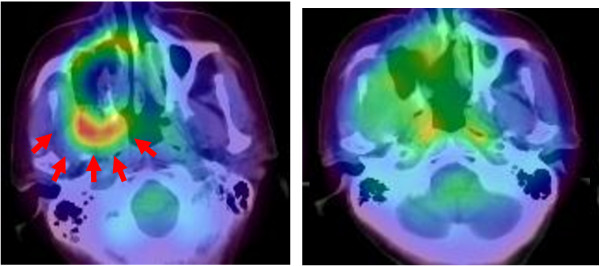
^**18**^**FBPA-PET study prior to and 7 months following the first BNCT treatment.A.** A 61 year-old female with residual maxillary adenoid cystic carcinoma (arrows) infiltrating into pterygopalatine fossa (T4N1M0) after maxillectomy, who was treated twice with BNCT using BPA followed by chemotherapy. **B.** Residual maxillary cancer and a regional lymph node metastasis were no longer evident at 42 months although bilateral multiple pulmonary metastases were detected at 18 months after the first BNCT treatment. The patient lived for 59 months following BNCT (*Applied Radiation and Isotopes,* 67:S37-S42, 2009).

**Figure 10:**
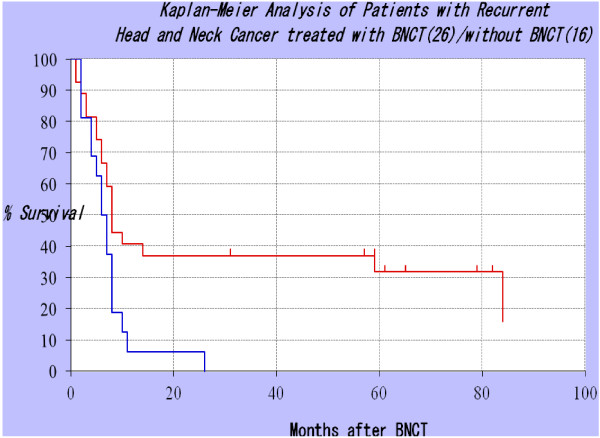
**Kaplan-Meier survival plots of patients with recurrent HNC treated by Kato et al. (6) with BNCT (26 cases, red line) and those who treated with other than BNCT (16 cases, blue line).** The outcomes for the 26 patients: Mean survival time: 33.6 months, 4-year Overall survival (OS): 37.0%, 6-year OS: 31.7%. Most of the 26 patients had either recurrent or far advanced cancers of the head and neck region and 15 (58%) had regional lymph node metastases and 6 had developed distant metastases. Nineteen of the patients had squamous cell carcinomas, 4 salivary gland carcinomas and 3 had sarcomas. All but one had received standard therapy and developed recurrent tumors for which there were no other treatment options.

**Figure 11:**
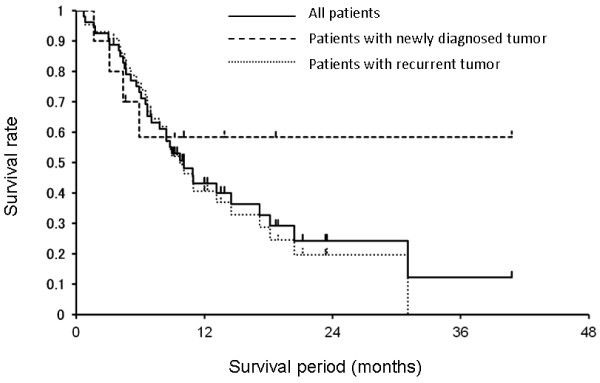
**Kaplan-Meier survival plots of patients with either newly diagnosed or recurrent tumors of the head and neck region, treated by Suzuki et al. with BNCT.** A total of 68 patients were treated. One and 2 year OS rates were 43.1% and 24.2% respectively. Thirty-three patients had squamous cell carcinomas (53%), 20 had adenocarcinomas (32%) and 11 (18%) had malignant melanomas.

The use of BNCT to treat patients with recurrent cancers of the head and neck region who had failed all other therapies was first initiated in 2001 by Kato and his co-workers in Japan [5] at the Kyoto University Research Reactor Institute (KURRI). The rationale for this was based on the ability of BNCT to deliver a large additional dose of radiation to the site of the tumor with a sparing of contiguous normal tissues. The first patient treated had a recurrent mucoepidermoid carcinoma of the parotid gland. Locoregional control was achieved and she died 7 years later of unrelated disease (Figure 8). Similar, clinically impressive results were seen in other patients treated by Kato et al. [5] and ^18^F-BPA and ^18^F-FDG PET scans from two patients are shown in Figures 9 and 4, respectively. Kato et al. survival data are summarized in the Kaplan-Meier plots shown in Figure 10. Based on these promising results two independent clinical trials were initiated in Japan by Kato and Suzuki and their respective teams to evaluate the safety and efficacy of BNCT in patients with a variety of malignancies of the head and neck region and these are summarized in Figures 10 and 11 and Table 4. A total of 68 patients with either newly diagnosed or recurrent, therapeutically refractory tumors were treated by Suzuki et al. between December 2001 and September 2007 (Figure 11). BPA and BSH, either alone or in combination, were used as the capture agents with either single or multiple applications of BNCT. Initially, BPA was administered i.v. at a dose of 250 or 500 mg/kg over 1–2 h, followed by BNCT using an epithermal beam within 15 min after termination of the infusion of BPA. Beginning in June 2004, BPA was infused at a dose of 500 mg/kg over 3 h at a rate of 200 mg/kg h for the first 2 h, and then at a rate of 100 mg/kg h for the final hour during which time the patient received BNCT. For patients who had received both BSH and BPA, epithermal neutron irradiation was initiated 12 h after termination of the infusion of BSH and one hour after that of BPA. The duration of BNCT was adjusted so that the majority of the cases the maximum radiation dose to the surrounding skin and normal tissue would be <10-12 Gy_w_.

**Table 4:** BNCT clinical trials using epithermal neutron beams for patients with recurrent or untreated unresectable head and neck cancer

**Medical institution**	**Neutron source**	**Treatment dates**	**Tumor type & No. of patients**	**Boron compound**^**a**^	**Clinical outcome**	**Ref.**
Osaka University, Oral & Maxillofacial Surgery II, Osaka, Japan	KURR (Kyoto University Research Reactor, Osaka, Japan) or JRR-4, Japan Atomic Energy Agency, Tokai Research Establishment, Ibaraki, Japan	2001-2007	26 rH&N	BSH 5 g & BPA 250 mg/kg in 1 h Or BPA 250–500 mg/kg in 1–2 h	MeST: 7.9 mos. post BNCT	[5,6], This Paper
					PR: 39%	
					CR: 46%	
					2 & 4 y OS: 37%,	
					6 y OS: 32%	
Osaka Medical College, Osaka, Japan	KURR	2005-2008	6 rOC	BPA 500 mg/kg (200 mg/kg h × 2 h; 100 mg/kg h × ~1 h during irradiation)	PR: 67%	[113-115]
					CR: 17%	
Kyoto University Research Reactor Institute (KURRI), Kyoto University, Osaka, Japan	KURR	2001-2007	49 rH&N	BSH & BPA 250–500 mg/kg	PR: 29%, CR: 28%	[116,117] This paper
			13 urH&N			
					MeST: 10.1 mos. (n = 53)	
					2 y OS: 24%	
Kawasaki Medical School, Kurashiki, Japan	KURR or JRR-4	2003-2007	10 rSqCC	BPA 500 mg/kg (200 mg/kg h × 2 h; 100 mg/kg h × ~1 h during irradiation)	PR: 35%	[4,118,119]
			7 rnSqCC			
			3 nSqCC		CR: 55%	
					2 y OS: 32.3%	
	JRR-4	2005-2008	10 H&N MM	BPA 500 mg/kg	PR: 50%	[120]
					CR: 40%	
Helsinki University Central Hospital, Helsinki, Finland	FiR-1, VTT Technical Research Centre, Espoo, Finland	2003-2008	24 rSqCC	BPA 400 mg/kg in 2 h	PR: 31%	[7]
					CR: 45%	
			6 rnSqCC			
					MeST: 13 mos. post BNCT	
					2 y OS: 30%	
		2010	1 ur PDC	BPA 400 mg/kg in 2 h	CR: 1/1	[121]
				BNCT + Chemo-RT (IMRT + SRT) with cetuximab and cisplatin		
Taipei Veterans General Hospital, Taipei, Taiwan	THOR, National Tsing Hua University, Hsinchu, Taiwan	2010-2011	10 rH&N	BPA ~450 mg/kg (180 mg/kg h × 2 h; 90 mg/kg h × ~1 h during irradiation)	PR: 40%	[122,123]
					CR: 30%	

^a^This column also indicates treatment in cases of multi-modality treatment.

Abbreviations: *rH&N* recurrent head and neck cancer, *rOC* recurrent oral cancer, *ur* unresectable, *rSqCC* recurrent squamous cell carcinoma, *rnSqCC* recurrent non-squamous cell carcinoma, *nSCC* non-squamous cell carcinoma (naïve), *H&N MM* mucosal melanoma of the head and neck, *PDC* poorly differentiated carcinoma, *XRT* external beam radiation therapy (photons), *IMRT* intensity modulated radiation therapy, *SRT* stereotactic radiation therapy, *PR* partial response, *CR* complete response, *2 y OS* 2-year overall survival, *MeST* median survival time, given in months.

### Patient population and treatment results

All of Suzuki’s patients had unresectable advanced or recurrent cancers of the head and neck region. Forty-nine patients (79%) had recurrent tumors and 13 (21%) had newly diagnosed unresectable tumors. Among the 49 patients, 39 (80%) had previous chemotherapy, 41 (84%) had previous surgical resection, and 36 (73%) had previous radiotherapy. Treatment sites included the oral and nasal cavities, paranasal sinuses and neck. Forty-two of the 62 patients received BNCT once, 17 received it twice, two received it three times, and one received it five times. Based on CT or MR imaging of 57 patients who had any target lesions, 16 showed a CR, and 17 had a PR for an overall response rate (CR + PR) of 58% at 6 months. MeST of 53 evaluable patients was 10.1 months from time of BNCT, and 1- and 2- year OS rates were 43% and 24%, respectively. The one year survival for newly diagnosed unresectable tumors was 58% and for those with recurrent tumors it was 41%. The MeST and median progression free survival (PFS) time and 1-year PFS rate for patients with recurrent tumors were 9.7 months, 5.1 months and 5%, respectively. The best 1-year OS was seen in 6 patients with recurrent adenocarcinomas (100%), followed by 6 with melanoma (60%) and in 29 with squamous cell carcinoma (26%). The major acute grade 3 or 4 BNCT-related toxicities were hyperamylasemia, fatigue, mucositis/stomatitis and pain, all of which were manageable. The most serious complications were carotid artery hemorrhage in 3 patients, two of whom died from rupture of an infected carotid artery, which had been invaded by tumor. In summary, the overall response rate and survival rate in both studies were comparable to those obtained with chemotherapy and re-irradiation, but in the majority of patients this was accomplished in *one* single BNCT treatment. One significant limitation of the approach used here was that deep-seated tumors could not receive a sufficient dose due to the limited depth of penetration of epithermal neutrons and, therefore, BNCT should be limited to more shallow tumors.

### Study carried out in Finland

A third clinical trial, carried out in Helsinki Finland, recently has been reported by Kankaanranta et al. [7]. A total of 30 patients with inoperable, locally recurrent cancers of the head and neck region were treated with BNCT. The majority (24 patients) had squamous cell carcinomas, and 14 of these were staged as T4, N0. They all previously had surgery and conventionally fractionated photon irradiation with or without chemotherapy. Patients were evaluated prior to BNCT by means of ^18^PET using ^18^ F-BPA, whenever possible, and in order to be treated by BNCT the tumor to contralateral normal tissue ratio had to be at least 2.5:1. Patients who were treated with BNCT received two treatments at 3 to 5 week intervals. BPA-fructose (400 mg/kg b.w.) was infused i.v. for 2 h prior to neutron irradiation, which was carried out at the Finnish BNCT facility in the outskirts of Helsinki. Neutron irradiation was performed via 2 ports; each with a median beam time of 18.6 min. Cetirizine hydrochloride was administered orally prior to irradiation and dexamethasone (10–15 mg/d) afterwards, in order to alleviate radiation related edema. More detailed information relating to other treatments that these patients received is provided in Kankaanranta et al.’s recent report [7]. Twenty-nine patients were evaluable with a median PFS of 7.5 months, of which 22 (76%) responded, 6 (21%) had stabilization of tumor growth for 5.1 and 20.3 months and 1 progressed. Two-year OS was 30% and PFS was 20% and 27% of the patients had no evidence of recurrent disease at 2 years. Mucositis and oral pain were the most common grade 3 adverse events, followed by fatigue, bone necrosis in 3 patients and soft tissue necrosis in one. Based on these results it was concluded that BNCT was effective for the treatment of inoperable locally recurrent, previously irradiated patients with head and neck cancer. Some responses were durable, but usually progression was common, most frequently at the site of the previously recurrent tumor.

### Study carried out in Taiwan

Finally, ten patients with recurrent, late stage cancer of the head and neck region have been treated at the National Tsing Hua University THOR reactor in Taiwan using BPA as the boron delivery agent. At the time of this writing 3 of the 10 patients have had complete regressions at the site of treatment [122].

The clinical results described above clearly have demonstrated efficacy, something that has been less convincingly demonstrated in patients with high grade gliomas. This is hardly surprising since it took a large randomized EORTC trial [124,125] with 590 glioma patients to convincingly demonstrate that the administration of temozolomide concomitantly with radiation therapy produced a significant increase in overall median survival compared to radiation therapy alone and this was only 2.5 months (14.6 versus 12.1 months)!

## Conclusions

What then is the future of BNCT? It probably lies in filling a niche for those malignancies, whether primary or recurrent, for which there is no effective therapy. What are some of the advantages of BNCT? *First*, it has the ability to selectively deliver a high radiation dose to the tumor with a much lower dose to surrounding normal tissues. This is an important feature that makes BNCT particularly attractive for salvage therapy of patients who have been treated to tolerance with photon irradiation. *Second*, it has the potential to more effectively target multicentric deposits of tumor than is possible with stereotactic radiosurgery of primary and metastatic brain tumors. *Third*, although it may be only palliative, it can produce striking clinical responses, as evidenced by the experience of several groups treating patients with recurrent, therapeutically refractory head and neck cancer [4-7,122]. Furthermore, as is evident from data compiled in Table 2 summarizing the clinical results obtained in treating patients with either primary or recurrent high grade gliomas, there has been significant progress in improving the clinical results. Specifically, increasing the dose of BPA and administering it over a longer time period (92–95) or combining BNCT with a photon boost, as has been carried out in Japan (80), have resulted in the best survival data obtained to date using BNCT to treat patients with gliomas.

Critical issues that must be addressed include: 1. Development of new low and high molecular weight boron agents and optimization of their delivery; 2. Obtaining approval for the clinical use of these new agents from regulatory agencies and carrying out biodistribution studies in patients prior to resection of their tumors; 3. Prioritizing collaborative efforts to compare and normalize dose prescriptions between centers, thus enabling studies on larger trial populations and perhaps facilitating multi-institutional or possibly randomized clinical trials; 4. Improvement of methods to determine the boron dose delivered to the residual tumor volume on both macroscopic and microscopic levels to enable more accurate tumor dose assessment. 5. Be prepared to compete with or complement new therapeutic approaches. In this review we have summarized the current status of BNCT as a treatment for high grade gliomas and recurrent tumors of the head and neck region and outlined problems that we believe are important to solve in order to move forward with BNCT as a treatment modality. It is up to investigators, both in basic and clinical research, to come up with solutions to these problems.

## Abbreviations

ABNS, Accelerator based neutron sources; ACNU, Aminochlorethyl nitrosourea; BMRR, Brookhaven Medical Research Reactor; BNCT, Boron neutron capture therapy; BNL, Brookhaven National Laboratory; BPA, Boronophenylalanine; BSH, Sodium borocaptate; CR, Complete response; CT, Computerized tomography; CTV, Clinical Target Volume; DVH, Dose volume histogram; EGFR, Epidermal growth factor receptor; EORTC, European Organization for Research and Treatment of Cancer; FCB, Fission converter beam; FDG, Fluorodeoxyglucose; GBM, Glioblastoma multiforme; Gy_w_, Weighted Gray; KURRI, Kyoto University Research Reactor Institute; L/N, Lesion to normal brain; MeST, Median survival time; MGH, Massachusetts General Hospital; MiMMC, Multi-Modal Monte Carlo; MIT, Massachusetts Institute of Technology; MITR, MIT Research Reactor; MoAb, Monoclonal antibody; MR, Magnetic resonance; MST, Mean survival time; MeST, Median survival time; MW, Megawatt; NCT, Neutron capture therapy; NV, Nanovehicle; OS, Overall survival; PDT, Photodynamic therapy; PET, Position emission tomography; PR, Partial response; TPS, Treatment planning system; VEGFR, Vascular endothelial growth factor receptor; WHO, World Health Organization.

## Competing interests

The authors declare no competing interests.

## Authors’ contributions

RFB: Had overall responsibility for the preparation of this manuscript. Wrote the Abstract, Introduction and Conclusions. Contributed to the section on “Boron Delivery Agents.” Contributed to the section on “Clinical studies of BNCT for brain tumors.” Rewrote the section on “Clinical studies of BNCT for head and neck cancer.” Compiled the references and integrated them into the text. MGHV: Wrote the section on “Boron delivery agents.” OKH: Wrote the section on “Neutron sources for BNCT.” WSK III: Wrote the section on “Clinical dosimetry and treatment planning” and contributed the related figures. Compiled the data and wrote the final versions of Tables 2, 3 and 4. KJR: Wrote the section on “Clinical dosimetry and treatment planning” and contributed the related figures. PJB: Wrote the section on “Clinical dosimetry and treatment planning” and contributed the related figures. FMW: Compiled the data and wrote the initial version of Tables 2 and 3. MS: Wrote the section “Clinical studies of BNCT for head and neck cancer” and contributed related figures. TA: Wrote the section “Clinical studies of BNCT for head and neck cancer” and contributed related figures. IK: Wrote the section “Clinical studies of BNCT for head and neck cancer” and contributed related figures. SK: Wrote the section on “Clinical studies of BNCT for brain tumors” and contributed related figures. All authors read and approved the final manuscript.
